# Obstructive sleep apnea event detection using explainable deep learning models for a portable monitor

**DOI:** 10.3389/fnins.2023.1155900

**Published:** 2023-07-14

**Authors:** Ángel Serrano Alarcón, Natividad Martínez Madrid, Ralf Seepold, Juan Antonio Ortega

**Affiliations:** ^1^School of Informatics, Reutlingen University, Reutlingen, Germany; ^2^Computer Languages and Systems, University of Seville, Sevilla, Spain; ^3^Computer Science, HTWG Konstanz, Konstanz, Germany

**Keywords:** obstructive sleep apnea, sleep apnea, portable monitor, deep learning, 1D-CNN

## Abstract

**Background:**

Polysomnography (PSG) is the gold standard for detecting obstructive sleep apnea (OSA). However, this technique has many disadvantages when using it outside the hospital or for daily use. Portable monitors (PMs) aim to streamline the OSA detection process through deep learning (DL).

**Materials and methods:**

We studied how to detect OSA events and calculate the apnea-hypopnea index (AHI) by using deep learning models that aim to be implemented on PMs. Several deep learning models are presented after being trained on polysomnography data from the National Sleep Research Resource (NSRR) repository. The best hyperparameters for the DL architecture are presented. In addition, emphasis is focused on model explainability techniques, concretely on Gradient-weighted Class Activation Mapping (Grad-CAM).

**Results:**

The results for the best DL model are presented and analyzed. The interpretability of the DL model is also analyzed by studying the regions of the signals that are most relevant for the model to make the decision. The model that yields the best result is a one-dimensional convolutional neural network (1D-CNN) with 84.3% accuracy.

**Conclusion:**

The use of PMs using machine learning techniques for detecting OSA events still has a long way to go. However, our method for developing explainable DL models demonstrates that PMs appear to be a promising alternative to PSG in the future for the detection of obstructive apnea events and the automatic calculation of AHI.

## 1. Introduction

PSG is the gold standard for detecting OSA (Mostafa et al., 2019; Kim et al., 2022). Its effectiveness is far from doubt. However, it has many widely known drawbacks, such as long waiting lists in hospitals, patients staying overnight in sleep laboratories with many sensors on their bodies, and the need for sleep clinicians during the study. In short, PSG requires a long time to be carried out and is economically expensive.

Alternatives to detect OSA have been appearing for some time (Collop et al., 2011). Some alternatives are intended to complement PSG to reduce patient waiting times, such as surveys (STOP-BANG) (Chung et al., 2016). At the same time, other alternatives claim to be an effective solution that can be used instead of polysomnography: PMs (Kirsch, 2013; Chang et al., 2020). Many PMs have been developed to detect OSA and are a booming technology for monitoring sleep disorders (Collop et al., 2011; Mendonça et al., 2019; McClure et al., 2020; Serrano Alarcón et al., 2021). However, practically all the devices have common objectives to perform the sleep test outside the sleep laboratory, to be as inexpensive as possible, to be sufficiently accurate, and to be as non-invasive as possible for the patient (Gjevre et al., 2011). Many solutions require a few physiological signals to determine whether the patient has OSA. The methods used to determine if the patient has this pathology are diverse. Despite this fact, one is currently the most widely used and promises the best results: classical machine learning and deep learning models (Thorey et al., 2019; Zemouri et al., 2019).

Deep learning algorithms generally detect sleep patterns, considering that, in most cases, they outperform machine learning algorithms (Cen et al., 2018; Mostafa et al., 2019; JeyaJothi et al., 2022). Specifically, deep learning avoids the inconvenience of having extensive knowledge in the specific field to extract the most relevant features (Bock et al., 2021; Zhang et al., 2021). There are several techniques for classifying biomedical time series, ranging from using shapelets to deep learning, including classical machine learning models (Bock et al., 2021). Artificial intelligence is taking its place among the most established techniques for generating more precise results and being easier to develop. There are a large number of studies that have used artificial intelligence algorithms to detect sleep apnea (Mostafa et al., 2019; Ramachandran and Karuppiah, 2021; JeyaJothi et al., 2022). Some studies assess the use of different architectures to test which one gives the best results when working with time series (Fawaz et al., 2019). However, not all scientific papers intend to use these DL algorithms in PMs to detect OSA. Only some of the publications focus on an essential aspect when trying to develop a medical device that is used in a real medical environment: the explainability of the model (Gaube et al., 2021). If there is something about which the use of machine learning algorithms raises doubts, it is sometimes difficult to determine why a model has made a decision and not another (Wang et al., 2020). This aspect becomes even more crucial when using these algorithms in real medical settings. Besides achieving an acceptable result to diagnose a particular pathology such as OSA, explaining why the model has made that decision is equally or more important. If a model is not interpretable enough, it can lead to legal consequences (Zemouri et al., 2019). Therefore, there should be a trade-off between the model accuracy and explainability/interpretability (Selvaraju et al., 2016). To explain the model decision, we can distinguish between local and global explanations. In the global explanation, the overall performance is analyzed. While in the local explanation, each example in the dataset is considered individually (Ivaturi et al., 2021). In this work, we are interested in knowing those regions of the signals most relevant for the model to make the prediction. Therefore, the technique known as Grad-CAM is chosen among the different visualization techniques currently used. When used with convolutional neural network (CNN), Grad-CAM uses the gradient information flowing into the last convolutional layer to assign importance values to each neuron for a given decision of interest (Gildenblat, 2017). Although Grad-CAM is widely used in image classification tasks, it also shows relevant results when classifying biomedical time series (Fauvel et al., 2021). Several existing implementations have been used and modified to implement Grad-CAM (Chollet, 2020). In addition, it must be presented clearly and concisely so the clinician can perfectly understand the prediction.

In the first stage of developing a PM to detect OSA, the problem of which data to train the models always arises. This fact occurs because, in the initial stages of the development of a PM, there is no patient data that the PM has collected. Therefore it must be decided which biomedical database to use. This fact is essential as the quality and extent of the data determine the success of training DL algorithms for detecting sleep patterns (Goldstein et al., 2020). The database should contain signals that best represent the physiological signals that the PM will measure in the future. This fact will be crucial in later stages since when the device is developed and ready for testing, the DL models used during the early stages will have to classify the biomedical signals collected by the PM. If these signals are not similar, the results will not be satisfactory. Along with the database to be used, it is also essential to determine which architecture or model will be used for detecting sleep apnea (Biswal et al., 2018). Literature analysis shows that CNNs are the most widely used architecture (Choi et al., 2018). It is also fundamental to define which methodology will be used to train the algorithms that work with time series. The most used are windowing and the use of the complete time series (Bock et al., 2021). In this work, the windowing technique will be used for various reasons that will be explained throughout this manuscript.

In short, this scientific work aims to develop DL models that detect obstructive sleep apnea events and estimate the AHI to be subsequently used on PMs in healthcare settings. A good interpretability of the model is needed, which will also be addressed throughout this manuscript. Numerous scientific publications comprehensively review different solutions for detecting OSA and other sleep disorders (Pathinarupothi et al., 2017; Chaw et al., 2019; Mendonça et al., 2019; Qian et al., 2021). Therefore, this work does not focus on reviewing existing solutions, but on developing a DL model so that a PM can subsequently use the model in a real clinical setting.

## 2. Materials and methods

**Figure 1:**
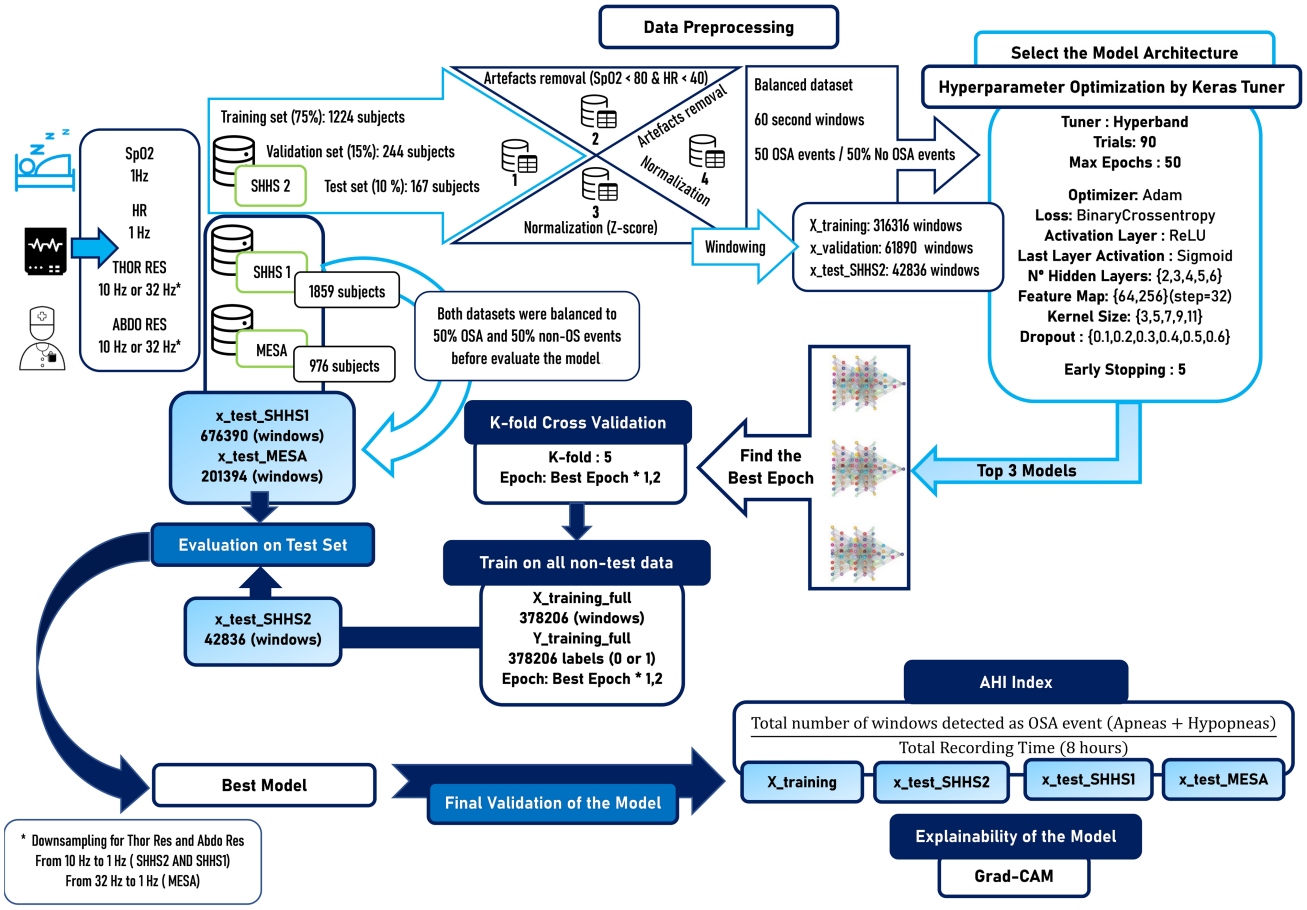
Workflow with the methodology applied in this scientific work. Several differentiated stages can be seen, such as the selection of the databases, the preprocessing of the signals, the balancing of the dataset to generate reliable results, the search for the best data model, a new training through cross-validation to verify the generalization of our model, the evaluation of the model in external datasets that are different from the one used for training and finally the calculation of the AHI together with the explainability of the model.

In this study, the set of signals with oxygen saturation (SpO2), heart rate (HR), thoracic respiratory effort (Thor-Res) and abdominal respiratory effort (Abdo-Res) were used to train and evaluate a DL model for OSA detection and AHI calculation. The workflow of the methodology used is summarized in Figure 1. The methodology applied in this study shows several distinct stages. First, the signals are obtained from the Sleep Heart Health Study 1 (SHHS1) (Zhang et al., 2018; Drzazga and Cyganek, 2021), Sleep Heart Health Study 2 (SHHS2) and Multi-Ethnic Study of Atherosclerosis (MESA) (Chen et al., 2015) databases. SHHS2 is used for training and validation. Data from SHHS1, SHHS2, and MESA are used for model testing. Four different datasets are generated from SHHS2 data to study the influence of artefact removal and signal normalization on the model results. The signals are divided into 60-s windows to subsequently balance the dataset, with 50% of the windows being apnea events and 50% non-apnea events that are randomly chosen. For the balancing, it is taken into account that the maximum number of sleep apnea windows is maintained. Subsequently, an optimizer is used to find the best architecture of the model using Keras Tuner. After the search for the best architecture, the three best models in terms of performance are selected, and new training is performed through cross-validation with *K*-fold = 5. The best-performing model is selected and re-trained on the whole dataset. Subsequently, the model is evaluated on SHHS2, SHHS1, and MESA. The best model is also used for the AHI calculation and for applying the Grad-CAM technique to facilitate the study of the decision taken by the deep learning model. Throughout the methods section, all these steps are explained in detail.

### 2.1. Dataset

The SHHS1 was performed from November 1, 1995, to January 31, 1998, and consists of raw polysomnography data from 5,793 patients. For its part, SHHS2 was carried out from January 2001–June 2003 and contained raw polysomnography data from 2,651 patients. The MESA is an NHLBI-sponsored 6-center collaborative longitudinal investigation of factors associated with the development of subclinical cardiovascular disease in 6,814 black, white, Hispanic, and Chinese-American men and women with baseline ages 45–84 years at baseline in 2000–2002. In subsequent studies, namely at MESA Exam 5 (2010–2013), several subjects participated in a sleep examination to collect (PSG). In total 2,060 PSG recordings were successfully collected (Chen et al., 2015).

After reviewing the literature it is not determined which dataset (SHHS1 or SHHS2) generates better results when feeding deep learning models. Therefore, it was decided to use SHHS2 for training the model since software and hardware updates were made for data collection, which may imply better data quality. In addition, the number of patients that the SHHS2 contains should be sufficient to train the model since it is better to have quality data than quantity as a general rule. Some studies have used both SHHS datasets, while others only SHHS1 or SHHS2 (Drzazga and Cyganek, 2021). In order to test the model with other external datasets, data from SHHS1 and MESA will be used to evaluate the model.

In biomedicine, obtaining sufficiently large and quality annotated datasets remains challenging (Zemouri et al., 2019). We have worked with four physiological signals in this work, that are explained in the next section. The DL model exposed in this work aims to be used with a PM in a natural clinical environment. However, the development of the device has yet to finish, and data cannot be obtained directly using it. Therefore, external datasets are needed since there is little or no evidence of using DL models trained with data from PMs at home (Kristiansen et al., 2021). In total, three datasets were used to develop and evaluate the models presented in this manuscript.

For training and validation of the models, patients from SHHS2 were used. Once the best-performing model was found, it was tested with patient data from SHHS1 and MESA to study its generalizability to new data. The number of patients selected and some of their characteristics can be seen in Table 1.

**Table 1:** Patient characteristics used for training, validation and testing of the models used in this scientific work.

Dataset	N° of subjects	Age	BMI	TST (min)	AHI	Male	Female
SHHS2 Train	1,224	67 ± 10	28 ± 5	398 ± 50	17 ± 15	44%	56%
SHHS2 TEST	163	67 ± 11	28 ± 5	398 ± 47	16 ± 13	44%	56%
SHHS1 TEST	1859	62 ± 11	28 ± 5	394 ± 46	15 ± 13	45%	55%
MESA TEST	976	69 ± 9	–	396 ± 55	22 ± 17	42%	58%

Age, BMI (body max index), TST (total sleep time) and AHI are shown as Mean ± Standard Deviation.

As can be seen in Table 1, there are similar proportions of women and men, which is essential to avoid bias in the classification task and develop as representative a model as possible. The patient data were randomly selected after discarding those patient data that contained a large number of missing values, a large number of artifacts or the sleep time was not longer than 300 minutes. The SHHS2 training dataset contains 994 patients with apnea and 230 without apnea. SHHS2 test contains 132 patients without apnea and 31 with apnea, while SHHS1 contains 1,518 patients with apnea and 341 without apnea, and MESA contains 863 patients with apnea and 113 without apnea.

#### 2.1.1. Signals

The clinical manifestation of sleep apnea presents variations in oxygen saturation levels, respiratory effort, and heart rate (Ramachandran and Karuppiah, 2021). A total of four signals are used for this project: SpO2, HR, Thor-Res and Abdo-Res by respiratory inductance plethysmography (RIP) (Mostafa et al., 2019). Sp02 and HR were originally sampled at 1 Hz for SHHS and MESA. Thor-Res and Abdo-Res were sampled at 10 Hz for SHHS and 32 Hz for MESA. In order to keep as much of the SpO2 signal (essential for hypopnea recognition) information as possible, to reduce the model training time and improve the visualizations at the model explainability stage, Thor-Res and Abdo-Res were downsampled to 1 Hz. In this way, all the signals used have the same sampling frequency, being essential to feed the algorithm that they all have the same amount of data points. The choice of 1 Hz as sampling rate has already shown promising results in the past (Kristiansen et al., 2021).

The combination of a small set of signals has shown promising results in the past (Haidar et al., 2018). A similar signal combination was used by (Biswal et al., 2018). Such work used raw airflow signals, respiration signals (chest and abdomen belts), and SaO2 with a recurrent convolutional neural network (RCNN). There are several reasons for selecting these four signals to train the model. First, this set of signals is the one the PM will collect in the future, with the difference that instead of measuring Thor-Res and Abdo-Res, the PM will collect signals through electrical impedance pneumography (EIP). A decision must then be made whether to use the combination of Thor-Res and Abdo-Res or only one of these signals. In addition, sleep apnea events detected by instantaneous heart rate (IHR) can be better verified using SpO2 signal, achieving better accuracy and precision (Pathinarupothi et al., 2017). The use of Thor-Res and Abdo-Res signals allows obtaining respiration airflow indirectly, thus avoiding the use of oronasal-airflow sensors that are invasive for the patient (Elmoaqet et al., 2020).

The recording duration of the patient’s physiological signals for all datasets (SHHS2, SHHS1, and MESA) used for the model development is 8 h (28,800 s).

#### 2.1.2. Apnea-hypopnea index

The AHI is considered the most relevant metric for diagnosing the existence and severity of sleep apnea, indicating the number of apneas per hour (Mostafa et al., 2019). The severity classification of obstructive sleep apnea has four distinct groups: physiological standard (AHI < 5), mild sleep apnea (5 ≤ AHI < 15), moderate sleep apnea (15 ≤ AHI < 30), and severe sleep apnea (AHI ≥ 30) (Piorecky et al., 2021). Some algorithms also implement the calculation of the AHI (Drzazga and Cyganek, 2021). The physiological signals used for this study are divided into 60-s windows. Therefore, if a window is detected as apnea or hypopnea, that window counts as an OSA event. As the entire set of windows that make up the physiological signals collected from the patient constitute 28,800 s, the calculation of the AHI could be done by applying Equation 1.


(1) $$AHI=\frac{Totalnumerofwindowsdetected\;as\;OSA\;event}{TotalRecordingTime\;\left(TRT\right)}\;$$

As shown in equation ( 1 ), the AHI is calculated using the TRT instead of the total sleep time (TST). Therefore, although this may lead to an underestimation of the severity of AHI, the model developed in this work does not calculate the start and end of sleep time. Therefore, it is not possible to use the TST. Despite this, TRT is considered a good approximation to TST for calculating AHI with PMs. When it comes to PM, AHI is usually expressed as the respiratory event index (REI) (Massie et al., 2018). REI is the number of apneas or hypopneas counted per hour by the device. However, the term AHI will continue to be used in this work to avoid confusion between terms.

### 2.2. Preprocessing

This scientific work aims to feed the deep learning model with raw signals and apply as little preprocessing as possible. Some works have this approach as their purpose (McClure et al., 2020). Generally, filtering, windowing, and sampling are the most common preprocessing techniques. It also includes the normalization or standardization of the data in signal preprocessing (Cen et al., 2018; Manoni et al., 2020). This work focuses on studying how large artifact removal and signal normalization affect the performance of various deep learning models for sleep apnea detection.

#### 2.2.1. Windowing

According to the American Academy of Sleep Medicine (AASM), sleep apnea is the cessation of airflow (equal or greater than 90%) for at least 10 s, while hypopnea is defined as a 30% fall in airflow for at least 10s (Berry et al., 2012; Drzazga and Cyganek, 2021). The recommended AASM criteria stipulate that airflow reduction for hypopneas should be associated with arousal or oxygen desaturation of at least 3%. This definition was used for the development of the models and the calculation of AHI. Alternatively, AASM also accepts that hypopnea can be defined by airflow reduction associated with an oxygen desaturation of at least 4% (Piorecky et al., 2021). Considering the above and that an OSA event can range from 10 to 40 s, a window period of 60 s is the most appropriate. Short OSA event periods may increase the likelihood of splitting the apnea event between several windows and thus underestimate or overestimate the AHI, depending on the duration of the event. On the other hand, using longer windows may result in multiple apnea events in the same window. In addition, there is evidence of no performance gain with durations longer or shorter than 60 s (Kristiansen et al., 2021). Moreover, the duration of this window is ideal for later visualization of the results using the Grad-CAM technique. The ultimate purpose of the model is to be used in an accurate portable monitor used by physicians. In this way, the physician can obtain more information about the apnea event if its duration is 60 s, thus being able to study the course of the signals. Notwithstanding the above, scientific papers in the literature using windows of 10s or even less also show promising results (Urtnasan et al., 2018; Elmoaqet et al., 2020; Tsouti et al., 2020).

#### 2.2.2. Artifacts removal

**Figure 2:**
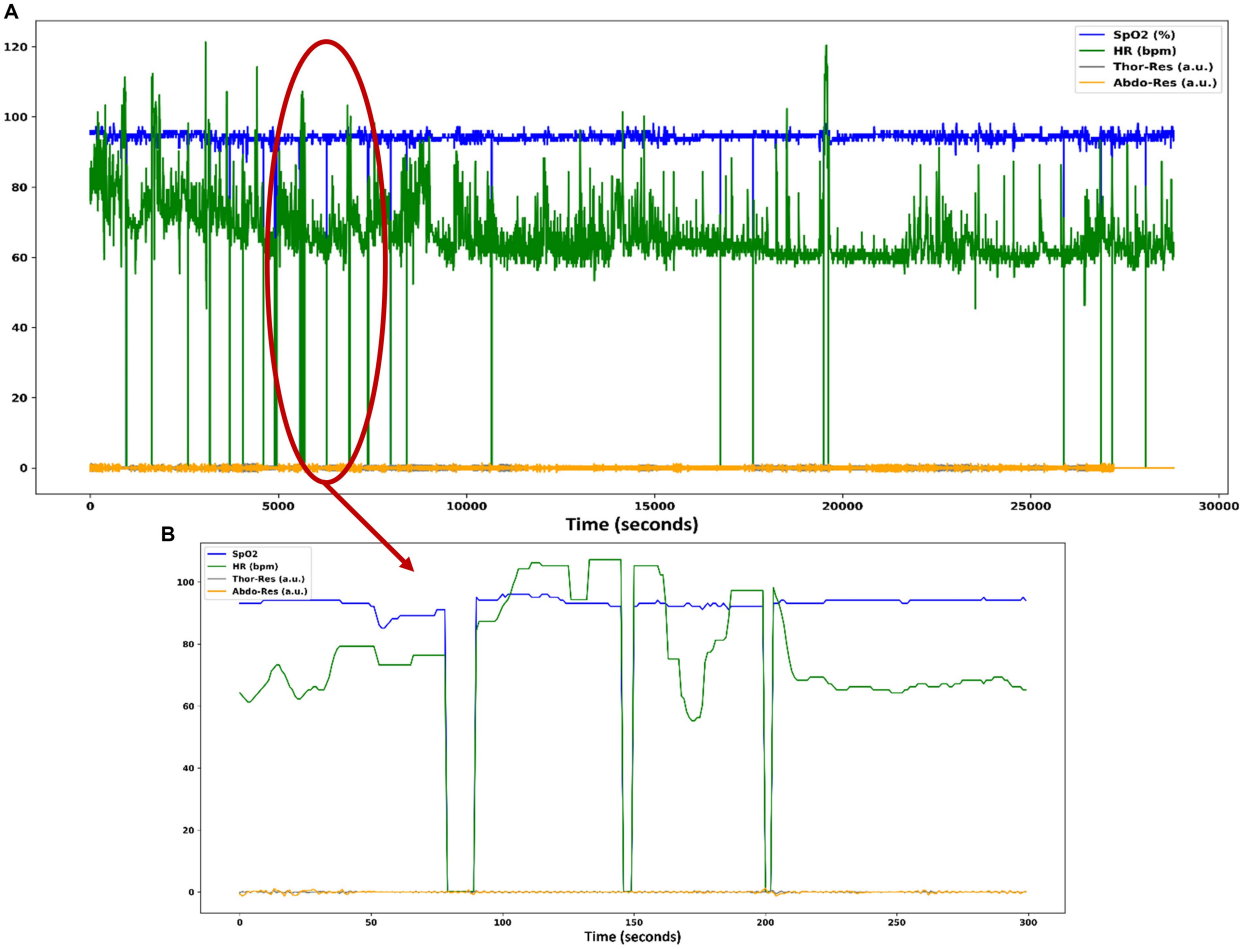
**(A)** Heavy artifacts in the SpO2 and HR signals of patient data in the SHHS dataset. **(B)** SpO2 and HR signals contain zero values for several seconds, causing numerous artifacts in these signals.

The occurrence of heavy artifacts is considered a fact to invalidate the reasoning analysis of the results (Moret-Bonillo et al., 2014). For this reason, the training and test data set was analyzed. After analysis of the data, it is observed that there are significant artifacts in the SpO2 and HR signals, as shown in Figures 2A,B.

**Figure 3:**
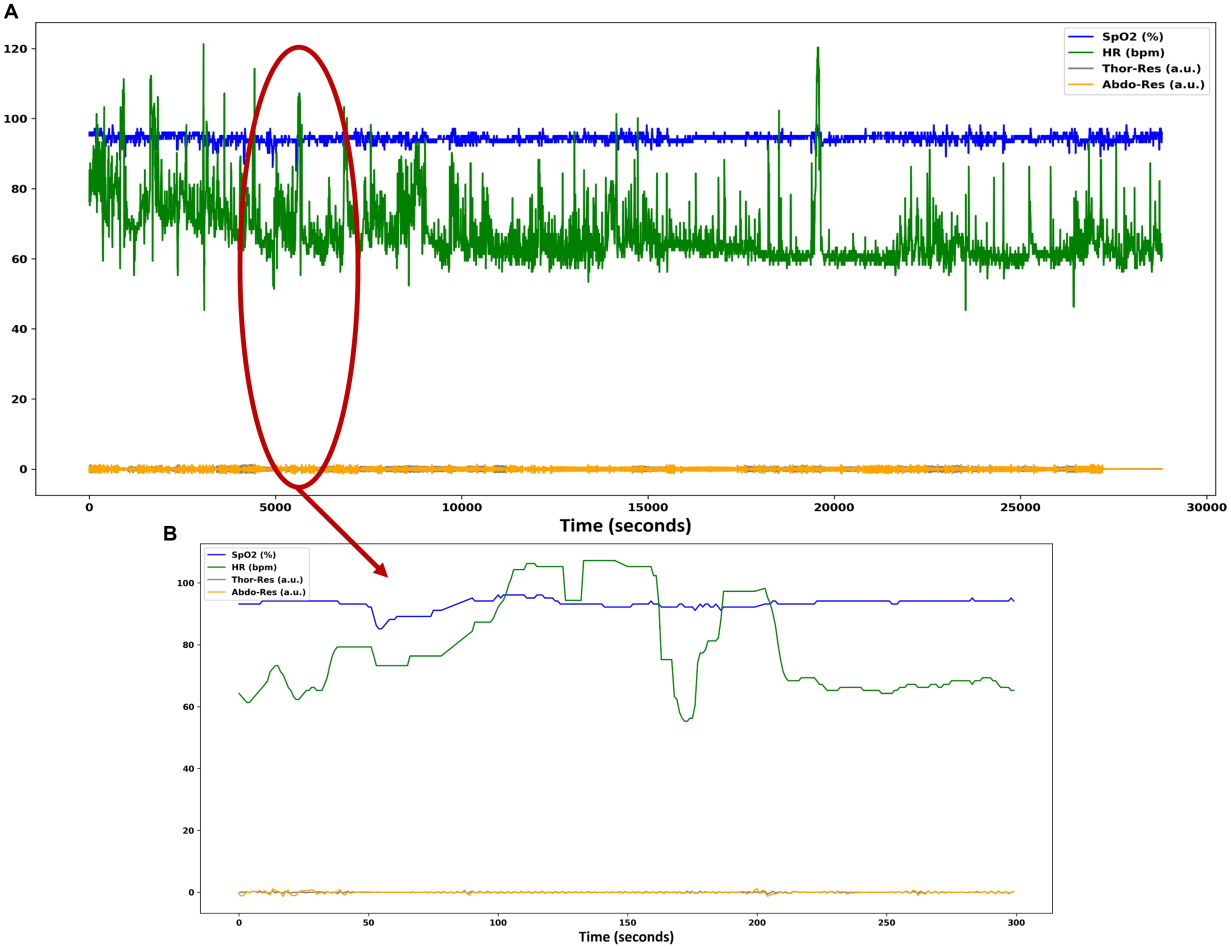
**(A)** Plot with the same set of signals as in Figure 1 where the heavy artifacts in the SpO2 and HR signals have been removed. **(B)** SpO2 and HR signals contain non-zero values as the artifacts have been removed.

Different datasets are generated for the subsequent training of the DL algorithms and to study the influence of artifacts on the classification. In two of the datasets created for the experiment, heavy artifacts are removed by interpolation when there are SpO2 data points below 80% and above 100%. For HR, values below 40 bpm and above 200 bpm are considered an anomaly. The signals after removing the artifacts look like Figures 3A,B.

#### 2.2.3. Normalization or standardization of the signals

In this work, the model is trained with different inputs. These input data can be used raw or normalized. As well as the study of the influence of the artifacts, the normalization of the signals is also relevant to the study of the model generalization. Therefore several datasets are generated and normalized. The normalized or standardized physiological signals range between 0 and 1. It is important to note that the data standardization is applied to the windows independently of the complete patient signal. This fact means that once the patient signals have been divided into 60-s windows, normalization is performed by applying Equation 2.


(2) $$z=\frac{X-\mu }{\sigma }\;\;$$

#### 2.2.4. Labeling

The signals contained in the SHHS and MESA also include annotations with the start and end of OSA events. The output of the developed DL model aims to classify the apnea events correctly. Therefore, those windows with apnea events greater than or equal to 10 s will be marked as an apnea event, regardless of their total duration. Physiological signals containing sleep apnea events are selected prior to windowing. The final selection can be seen in Table 1. The SHHS y MESA for AHI calculation, recognizes obstructive apneas with no oxygen desaturation threshold used and with or without arousal+ hypopneas with >30% flow reduction and > = 3% oxygen desaturation or with arousal.

### 2.3. Model architecture

Model building is the most significant difficulty when working with neural networks (NN). There is no guarantee that the number of hidden layers/units is optimal (Zemouri et al., 2019). Currently, several architectures usually give good results in many different fields, such as the recurrent neural network (RRN). Time series studies have shown that ResNet and CNN models achieve the best results in terms of classifying biomedical signals (Wang et al., 2017; Fawaz et al., 2019). However, RNNs are less frequently used than CNNs (Zemouri et al., 2019; Nassi et al., 2021). CNN is the most widely used neural network for classifying apnea events, so its capacity is beyond doubt (Mostafa et al., 2019). This work aims to achieve optimal results so the model can be used in a natural clinical environment. Some scientific papers suggest that PMs for clinical practice should have a sensitivity >82.5% (Collop et al., 2011). That would be the objective, in addition to reducing the complexity of the model and the minimal processing of the signals. CNNs seem the best option when working with biomedical time series because they work well with raw signals, require fewer computational resources, and require fewer data to obtain optimal performance (Wang et al., 2020; Bock et al., 2021). A variant of CNNs, the 1D-CNN, has been used for signal classification by many authors (Cen et al., 2018; Dey et al., 2018; Haidar et al., 2018; Urtnasan et al., 2018; McClure et al., 2020; Kim et al., 2022). Therefore a 1D-CNN model has been chosen as it has shown significant results in other studies and meets our requirements (Chang et al., 2020). The main feature of 1D-CNN is that kernels traverse input signals in one dimension, with either the width or the height (depending on how the input is oriented) of the kernel being configurable, but not both dimensions of the kernel being configurable as they would be in 2D-CNN.

Another reason why CNNs seem the best option to work with because they allow the implementation of visualization techniques to identify the regions of the signal that are most relevant for the model to make a certain prediction, such as Class Activation Map (CAM) and Grad-CAM (Fawaz et al., 2019).

In this work, four different datasets were used based on whether the artifacts of the SpO2 and HR signals were removed or the signal set was normalized. Based on this, four models were generated and trained.

The features of the proposed models are similar. All models contain the same pattern for the layers: a convolutional layer and a batch normalization layer that are used to improve the speed, performance, and stability of the neural network (Kim et al., 2022). Additionally, activation functions and regularization techniques are included to avoid overfitting the model. All the features of the layers of the chosen model are finally exposed in Section “Results”.

Some publications use trial and error techniques to choose the best model (Dey et al., 2018). However, for the selection of the best architecture of this model, a more engineering approach is used with the use of a hyperparameter optimization framework such as Keras Tuner.

#### 2.3.1. Hyperparameter setting

The architecture-level parameters, called hyperparameters, are among the most relevant tasks when working with DL algorithms. Despite the large number of publications that currently use DL models for sleep apnea detection, there is no standard for fine-tuning the model hyperparameters. In most cases, it is decided to modify the hyperparameters by hand and retrain the model repeatedly. However, this should be different, and a more empirical approach should be sought (Chollet, 2021). There are some publications in which a method is developed based on certain algorithms for an optimal configuration of the hyperparameters (De Falco et al., 2018). Despite this, it is a challenging task. For this reason, Keras Tuner is used to choose the best hyperparameters for our search space (see Table 2) (O’Malley et al., 2019).

**Table 2:** Search space for the search of the best hyperparameters by the optimizer.

Hyperparameters	Search space
Tuner	Hyperband
Number of hidden layers	{2,3,4,5,6}
Feature map	{64,96,128,160,192,224,256}
Kernel size	{3,5,7,9,11}
Dropout	{0.1,0.2,0.3,0.4,0.5}
Layer Activation (convolutional layer)	ReLU
Last-layer activation	Sigmoid
Optimizer	Adam
Loss	Binary Crossentropy

Keras Tuner offers several different tuners. For training our model, we opted for Hyperband (Li et al., 2018). The main operation of Hyperband is that it takes random samples of all hyperparameter combinations and does not run the full training and evaluation set. It trains the model for a few epochs with a set of hyperparameter combinations and selects the best candidates based on the results of these few epochs. It is performed iteratively and the tuner runs the chosen candidates through the complete training and evaluation set. In this aspect, Hyperband is better than other tuners like RandomSearch that perform the complete evaluation in each iteration. The Bayesian Optimization tuner was discarded as its operation is sometimes similar to a black box.

Regarding the set of hyperparameters, this search space has been established based on the results of other publications (Mostafa et al., 2019; Kristiansen et al., 2021; Ramachandran and Karuppiah, 2021). Specifically, the kernel size is relatively small because the number of data points per window is limited (60 s). On the other hand, the activation function chosen is rectified linear unit (ReLU) for the convolution layers and sigmoid for the activation layer of the last layer. These activation functions have been chosen because ReLU has demonstrated its good performance in numerous fields, specifically classifying OSA events (Chollet, 2021). For binary classification (two output classes), models are recommended to be terminated with a dense layer with a unit and a sigmoid activation (the model output should be a scalar between 0 and 1 that encodes a probability). Regarding the loss function, it is best to use the binary cross-entropy loss function as it is usually the best choice when developing models that output probabilities. For this reason, these hyperparameters are not searched using the tuner (Chollet, 2021). The Adam optimizer is chosen as it is widely used and has shown promising results in classification tasks.

#### 2.3.2. Training and evaluation

The data preprocessing and model training were carried out in a jupyter notebook application using Python language (version 3.9.7). The Python deep learning API Keras, which works on Tensorflow (version 2.8.0), were used to develop the models. The scientific computing library NumPy (version 1.21.5) and the machine learning library Scikit-Learn (version 1.0.2) were used for data processing tasks and model evaluations. The Workstation used to carry out the experiments comprises an Intel i7-11700K 3.60GHz processor, 32GB RAM, and an Nvidia GeForce RTX 3090 24GB Graphics Processing Unit (GPU).

The SHHS2 dataset was split into a training set (70%), a validation set (20%), and a test set (10%). From this dataset, four new datasets were generated. Four different training sessions were carried out, depending on the training dataset used. A dataset without artifact removal and without standardization: training dataset 1 (TrainDat1). Another dataset with artifact removal and without standardization: training dataset 2 (TrainDat2). Another dataset without artifact removal and with standardization: training dataset 3 (TrainDat3). Lastly a dataset with artifact removal and standardization: training dataset 4 (TrainDat4).

These four data sets and applying only some processing tasks are intended to evaluate the model to determine with which dataset the model yields the best results. The tuner Hyperband was used to search for the best set of hyperparameters. For this, a batch size of 1,024 was established since the batch size should not be treated as a tunable hyperparameter for validation set performance. Some studies suggest that the batch size should be long enough to be supported by hardware resources (Shallue et al., 2018; Godbole, 2023).

**Figure 4:**
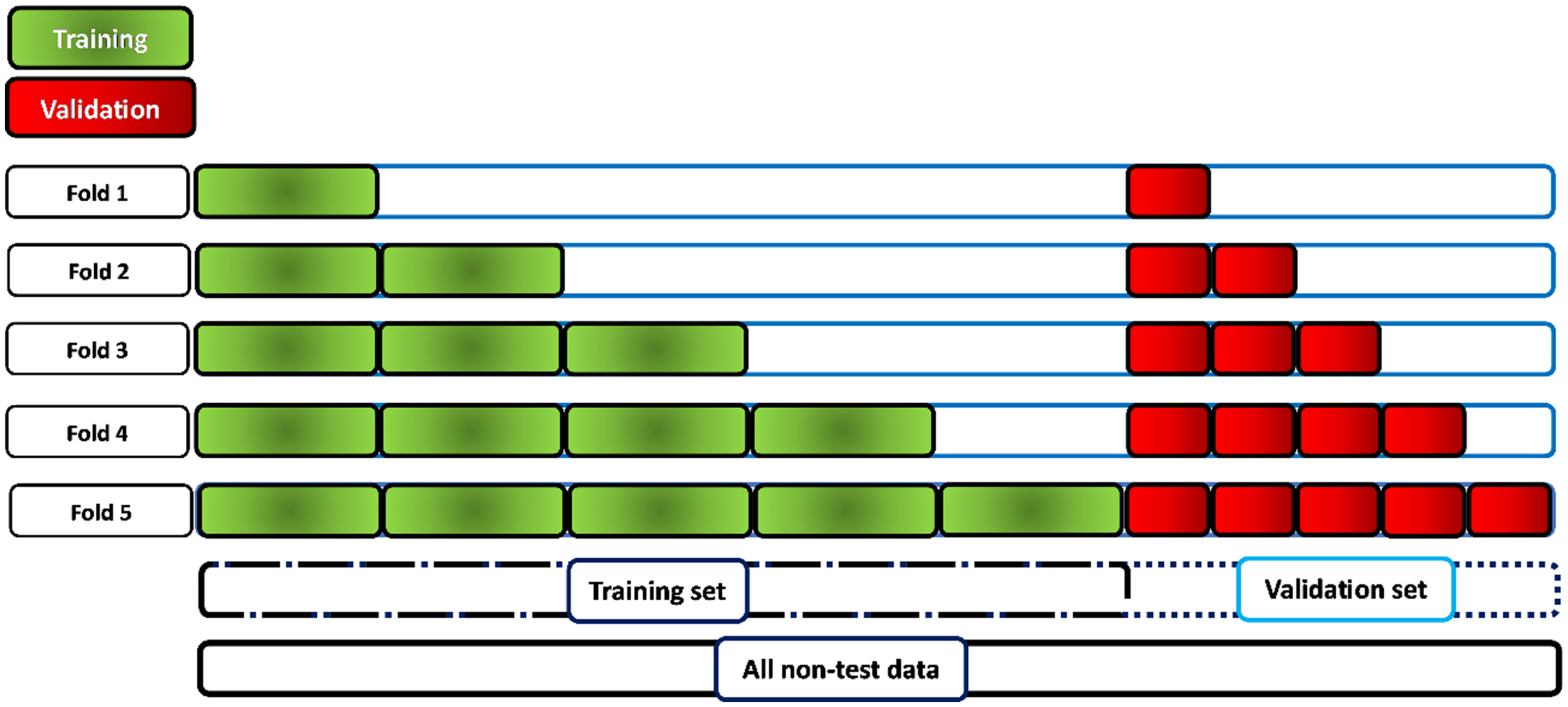
Methodology for the application of cross validation for the evaluation of the best model. When working with 60-s windows, it is not possible to perform cross-validation on the whole dataset, as there would be windows belonging to the same patient in both the training and validation sets. Therefore, the training and validation sets are increased by approximately 20% in each fold (5 times) in the training and validation sets with patient data independent of each other.

The metrics used to validate the model were binary crossentropy such as loss, accuracy, sensitivity, specificity, area under the curve (AUC), and precision. The tuner was configured not to train models older than 50 epochs. There is controversy over Hyperband determination of the number of models to train. According to (Gildenblat, 2017), one iteration will run approximately max_epochs * (math.log(max_epochs, factor) ** 2) cumulative epochs across all trials. However, according to (Lamberta, 2017), Hyperband determines the number of models to train in a bracket by computing 1 + log(max_epochs, factor) and rounding it up to the nearest integer. The maximum number of epochs established during the search for the best hyperparameters for the model was 50. The early stopping optimization technique was also used to create a callback to stop training after reaching a specific validation value for more than five epochs. After completing the search for the best hyperparameters by Keras Tuner, the three models that obtained the best validation accuracy value were selected. These models were retrained again to find the optimal epoch value from which the best value for loss validation is obtained and overfitting begins. After knowing the best value for the epoch, the model is retrained with a few more epochs of the best epoch by using cross-validation with a k-fold equal to five. As the physiological signals of the patients were divided into windows for the division of the data set into training, validation and test data, it is not possible to use all the available data for cross-validation, since the model would be trained and evaluated with windows that belong to the same patient. Therefore, the methodology shown in Figure 4 is applied. For the training and validation set, a proportion of windows is selected for the first fold, twice as many windows for the second, and so on for the rest of the folds until the entire data set is covered. In this case, the entire data set comprises the data for training and validation.

To facilitate the explanation of the results obtained by the deep learning models, the model trained with TrainDat1 is called Model 1. The model trained with TrainDat2 is called Model 2, the model trained with TrainDat3 was named Model 3. Lastly the Model 4 was trained on TrainDat4.

### 2.4. Explainability of the model

In addition to the difficulty in choosing the appropriate model architecture, the interpretation of the results obtained is equally relevant, where the concept known as the “black box” is fundamental to avoid in medicine. Model explainability is crucial when using DL models in the clinical setting. For this fact, the chosen neural network is a CNN with a Global Average Pooling (GAP) layer. The advantage of using this architecture and this layer is that the neural network can retain the remarkable localization ability until the final layer. In this way, using Grad-Cam, the most crucial signal region of the input can be discriminated (Zhou et al., 2016). The GAP unit receives the convolutional feature map as input and generates the spatial average of each feature map (Vijayarangan et al., 2020).

## 3. Results

### 3.1. Performance of the models after training

The number of models executed and trained by the Keras Tuner was 90 before finding the models that yielded the best results. The execution time for all the models trained and evaluated was between 8 and 10 h. The results obtained after training and their evaluation in SHHS2, SHHS1, and MESA are found in Table 3. It can also be seen in Supplementary Table S4, the results after testing the models on unbalanced data sets with a ratio of 1:3 with a lower number of apnea events.

**Table 3:** Overall results to evaluate the different 1D-CNN models trained on the balanced test datasets (SHHS1, SHHS2 and MESA).

	Model 1			Model 2			Model 3			Model 4		
Dataset	SHHS2	SHHS1	MESA	SHHS2	SHHS1	MESA	SHHS2	SHHS1	MESA	SHHS2	SHHS1	MESA
Accuracy	72.5	64.0	66.3	72.3	66.1	66.0	83.8	68.1	74.1	84.3	70.0	74.5
Loss	0.56	0.74	0.66	0.60	0.76	0.76	0.37	0.75	0.66	0.36	0.64	0.63
Sensitivity	54.4	44.9	50.1	56.9	45.8	52.3	80.5	46.0	71.6	82.5	53.7	76.0
Specificity	90.6	83.0	81.8	87.8	86.4	79.6	87.0	90.3	76.7	86.0	86.3	72.8
Precision	85.4	72.5	73.6	82.4	77.1	71.9	86.1	82.6	75.5	85.5	79.6	73.7
AUC	82.6	69.7	74.3	81.7	74.5	73.3	92.0	77.3	81.1	92.1	77.7	80.8

**Table 4:** Set of the best hyperparameters of the Model 4.

Tuner	Hyperband
Number of hidden layers	6
Feature map – 1st hidden layer	128
Kernel size – 1st hidden layer	7
Dropout – 1st hidden layer	0.3
Feature map – 2nd hidden layer	192
Kernel size – 2nd hidden layer	5
Dropout – 2nd hidden layer	0.3
Feature map – 3rd hidden layer	224
Kernel size – 3rd hidden layer	3
Dropout – 3rd hidden layer	0.4
Feature map – 4th hidden layer	96
Kernel size – 4th hidden layer	7
Dropout – 4th hidden layer	0.2
Feature map – 5th hidden layer	256
Kernel size – 5th hidden layer	9
Dropout – 5th hidden layer	0.3
Feature map – 6th hidden layer	96
Kernel size – 6th hidden layer	9
Dropout – 6th hidden layer	0.5
Layer Activation (all convolutional layers)	ReLU
Last-layer activation	Sigmoid
Learning rate	0.001
Optimizer	Adam
Loss	Binary Crossentropy

In order to study the results, we must first evaluate the metrics obtained in SHHS2, as this is the dataset used to train the models. In view of this and starting with the accuracy, we can observe that the best results are obtained with Model 4. This fact makes sense since the model was trained on patient’s signals from the same dataset (SHHS2). The worst results are given by Models 1 and 2. This fact implies that those datasets with standardized data would give better results. As a counterpart to standardization, we have the elimination of artifacts. As we can see from the results in Table 3, the datasets whose data were filtered to remove artifacts do not perform as well as the models where standardization was applied. Generally, when dealing with binary classification, it is necessary to support the result in accuracy with other metrics as not all datasets are balanced, and the results obtained can be misleading. In this work, the datasets were balanced at 50% with windows and apnea and 50% windows without sleep apnea. This implies that the accuracy gives a robust idea of the generalization power of our models. However, sensitivity, specificity, precision and AUC are shown in Table 3. Considering the results obtained by Models 3 and 4 are very similar. However, there are slight differences. For the evaluation of the model on SHHS1, the metrics improve slightly with Model 4, which uses a dataset in which the removal of artifacts and standardization has been applied. This could indicate that SHHS1 contains a more significant number of artifacts than another dataset, and therefore the removal of artifacts positively affects the performance of the model. The same is for SHHS1 with Models 1 and 2, where the results are slightly better for Model 2. However, the differences must be more substantial to use artefact removal to improve model performance. Considering other metrics, such as specificity, the results are robust for all the models and the datasets used. This indicates that the trained models recognize normal or non-apnea events well. Looking at AUC, the results are good enough to discern between apnea and non-apnea events since the value for AUC is between 70% and 93% for practically all the datasets used. Therefore, after studying the results obtained and attending to all the metrics, it can be affirmed that Model 4 is the algorithm that works best for the detection of apnea events. Therefore it will be the model analyzed throughout this section. For this purpose, its architecture and its performance in the AHI calculation will be studied. The outputs will also be analyzed using the Grad-CAM technique. Table 4 shows the set of hyperparameters that constitute the architecture of Model 4. The architecture consists of six hidden layers with kernel values ranging from three through seven to nine. The set of hyperparameters for the other models consists of four hidden layers, which can be seen in the Supplementary Tables S1,S2,S3.

**Figure 5:**
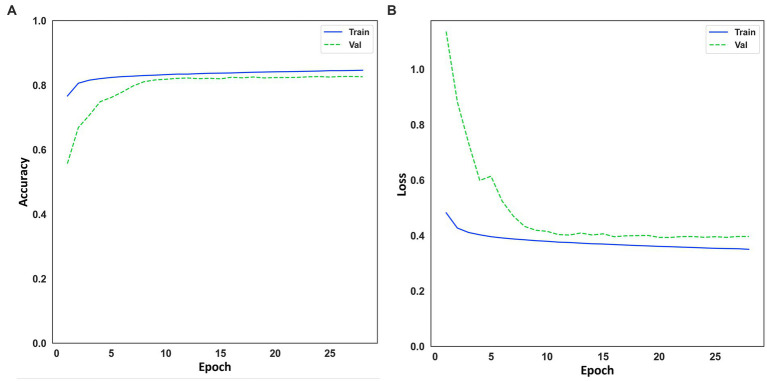
**(A)** Learning curve for accuracy on training and evaluation data. **(B)** Learning curve for loss on training and evaluation data.

Figure 5 shows the training curve for Model 4 throughout the cross-validation. Therefore, the curve is the different training runs (5-fold) overall. It can be seen that the model converges quickly, and there are no abrupt jumps. No overfitting is observed either. The fast convergence of the model can be explained by the high value of the batch size, which, as mentioned in section “Training and evaluation”, a high batch size was chosen that the hardware used for training could process. Having a look at Figures 5A,B for both accuracy and loss, it can be seen that the training and validation curves are similar, which implies good convergence of the model and hence appropriate generalization, where the model continuously learns from the input data. Considering this and looking at Table 3, it can be stated that Model 4 generalizes well for other datasets that the model has not used for training. Even though the results of the metrics are slightly worse, but still valid for the classification of OSA events. The mean value of the accuracy of the cross-validation application was 84.46 (±0.07) for the training set and 82.5 (± 0.64) for the validation set.

**Figure 6:**
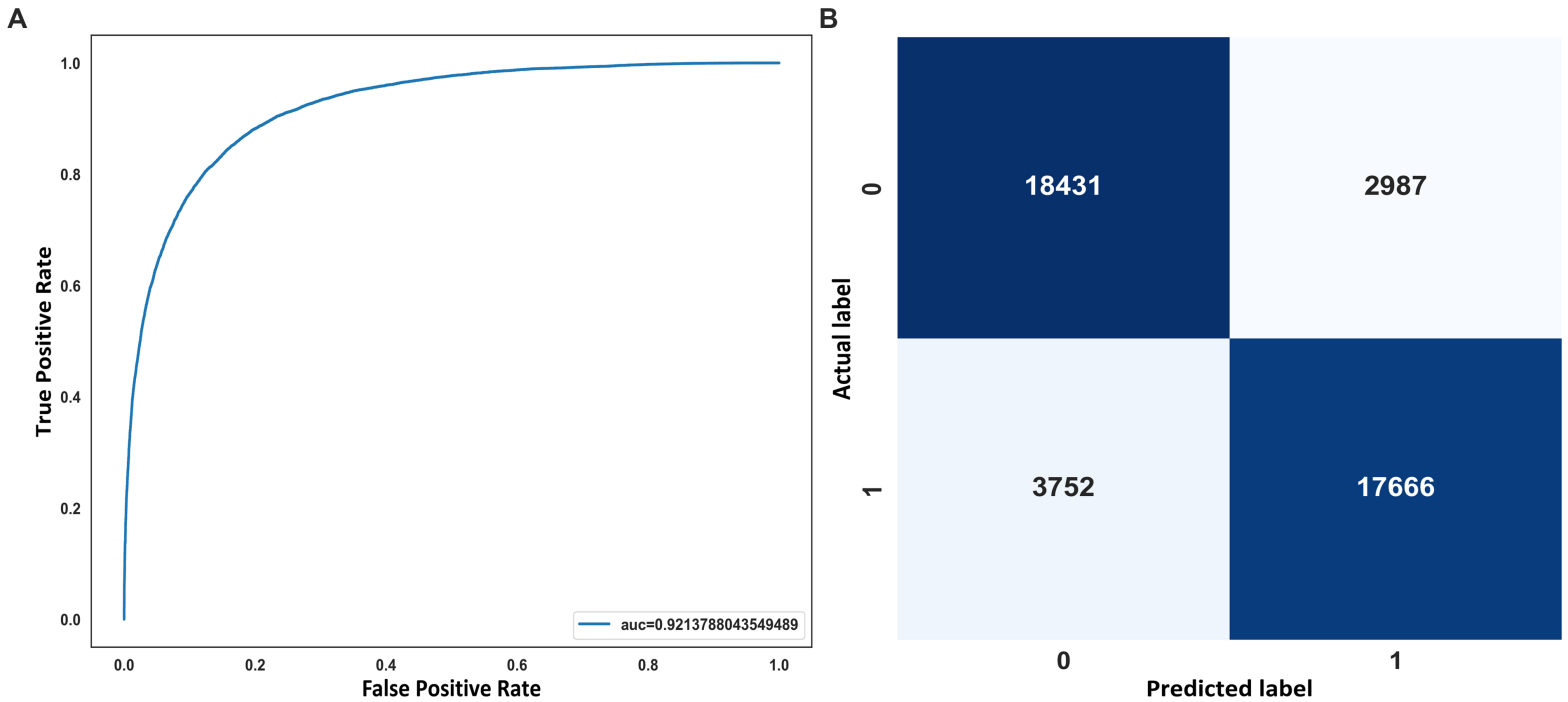
**(A)** ROC curve. **(B)** Confusion matrix on the SHHS2 test.

On the other hand, in Figure 6, it can also see the representation of the receiver operating characteristic (ROC) curve (Figure 6A) and the confusion matrix on the test set with 42.836 windows. The value of 92.1% of AUC indicates the model’s good performance when classifying OSA events. The confusion matrix shows how the vast majority of the 42,836 predicted windows were correctly classified (Figure 6B).

### 3.2. AHI estimation

**Figure 7:**
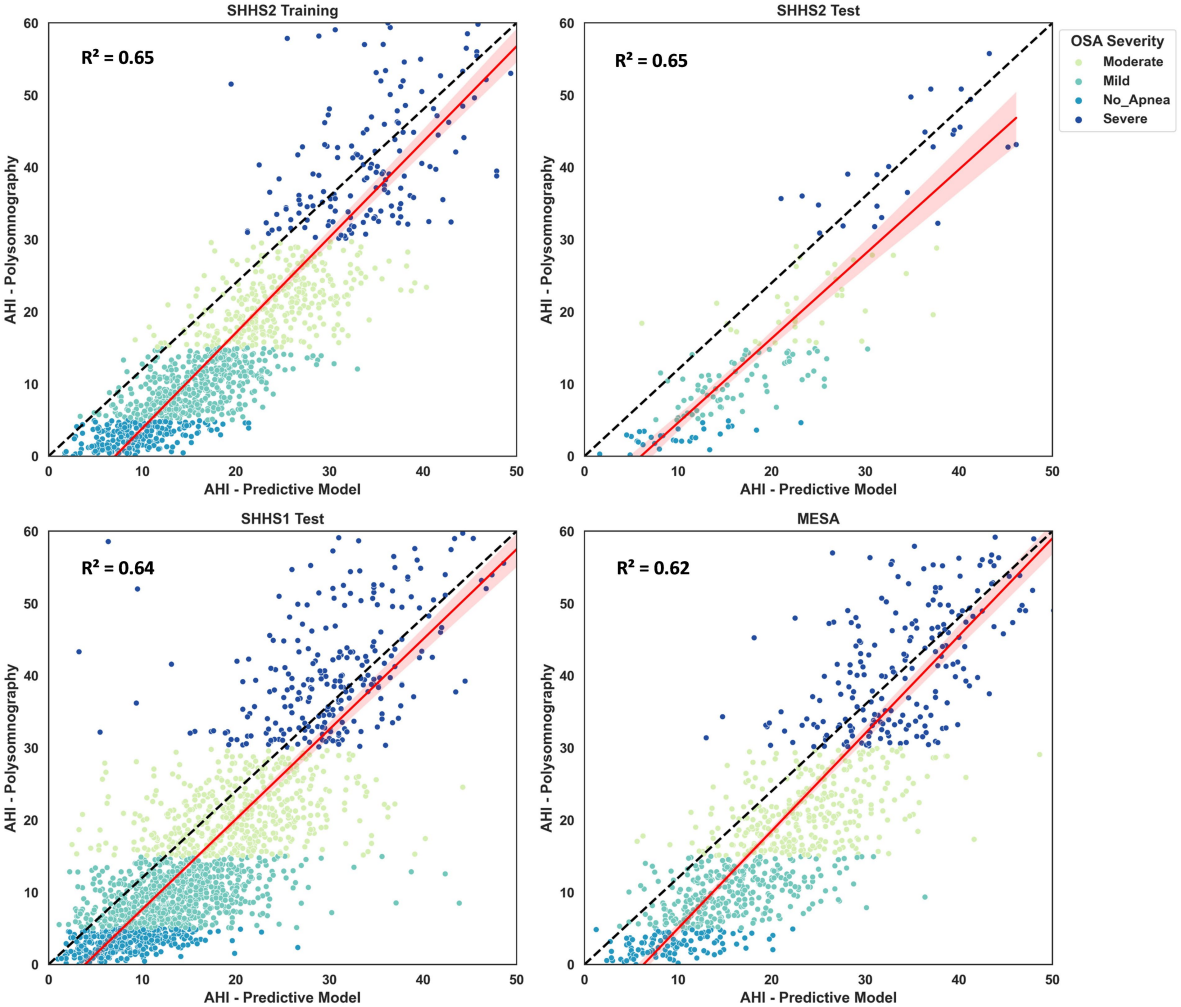
Scatter plots and regressions for the SHHS2, SHHS1 and MESA. The values for R2 are also shown in the different plots. The dash-dotted line indicates the identity line. Values are grouped according to apnea severity: no apnea, mild, moderate and severe apnea.

The AHI estimate was calculated for SHHS2 Training, SHHS2 Test, SHHS1 Test and MESA. The coefficient of determination (*R*^2^) was calculated to validate the AHI estimate. The confusion matrix was also generated to study correctly and incorrectly classified patients. As shown in Figure 7, the coefficient of determination is 0.65 for SHHS2 Training and SHHS2 Test, 0.64 for SHHS1 Test and 0.62 for MESA. This fact implies that Model 4 is able to discern between apnea and non-apnea events to account for AHI. Although there is an overestimation of AHI for all levels of apnea severity except for severe apnea, the *R*^2^ values show the potential of the model for both event classification and AHI calculation.

**Figure 8:**
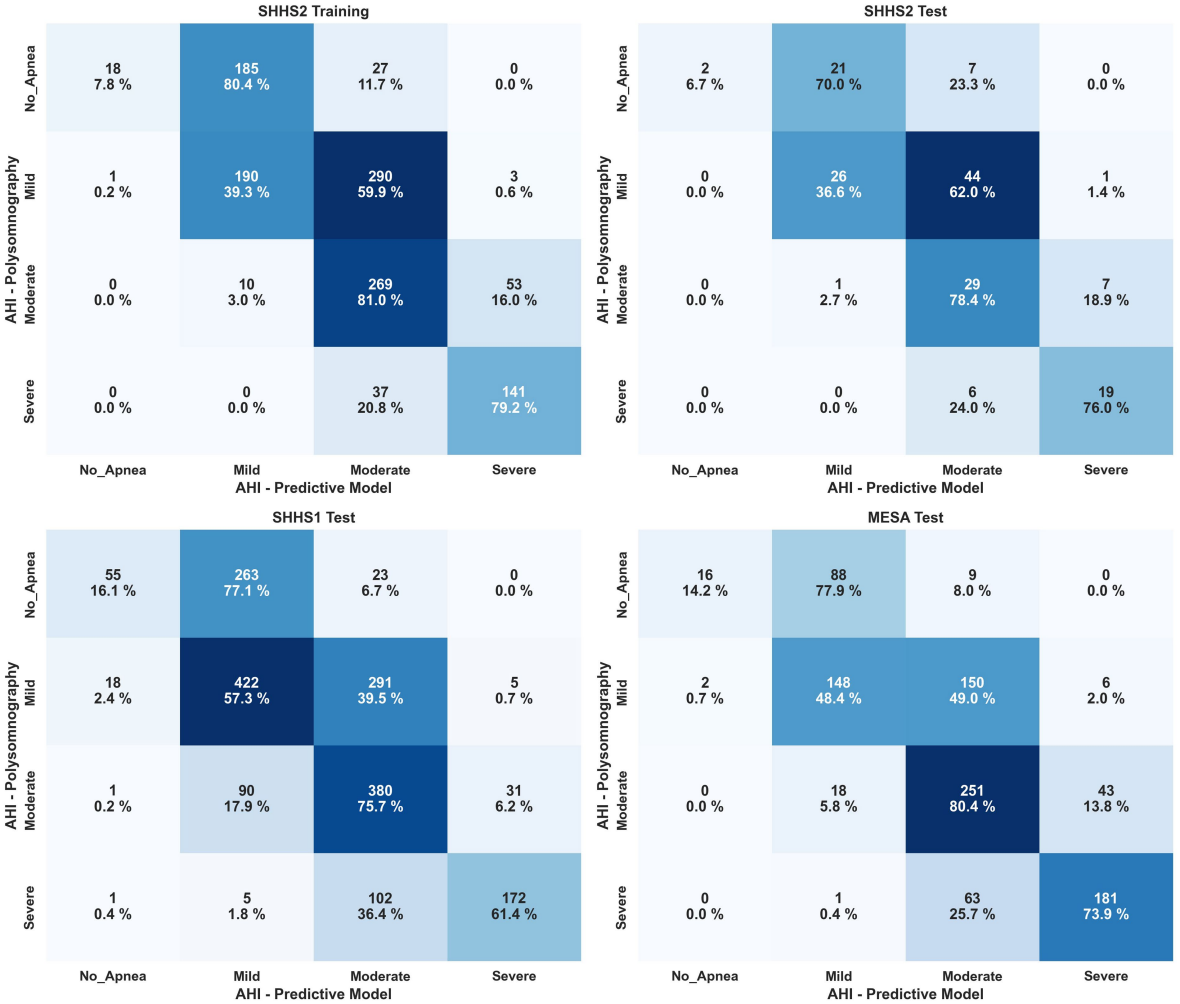
Confusion matrix for patient classification by OSA severity after AHI estimation for SHHS2, SHHS1 and MESA. Severity is shown by percentage in each cell and color scale, with darker colors being better.

Figure 8 shows the confusion matrix for classifying OSA severity for different SHHS2, SHHS1, and MESA. Based on the visualization, it can be stated that Model 4 is able to obtain good results for the classification of apnea patients within moderate or severe severity with hit rates of 72.6–78.8% on average for all datasets. However, the results could be better for the classification of non-apneic patients. The results are better for apnea patients with mild severity than those classified as non-apnea patients but still worse than for the moderate and severe severity grades. A fact that may explain the poor results obtained for classifying patients without apnea is that the range from zero to less than five events is the smallest of all existing ranges for classifying patients according to severity. Therefore, an overestimation by the model implies more significant errors in this classification segment. Table 5 shows the results generated by the model for the most common thresholds for the calculation of AHI. Weighted Cohen’s Kappa with linear weights has been applied as we worked with a multiclassification with ordinal values.

**Table 5:** Results for AHI 5 e/h, AHI 10 e/h, and AHI 15 e/h to evaluate the different 1D-CNN models trained on the training and test data sets (SHHS1, SHHS2, and MESA).

Dataset	SHHS2 training			SHHS2 test			SHHS1 test			Mesa test		
AHI (e/h)	AHI 5	AHI 15	AHI 30	AHI 5	AHI 15	AHI 30	AHI 5	AHI 15	AHI 30	AHI 5	AHI 15	AHI 30
Accuracy	82.6	73.0	92.4	82.8	67.5	91.41	83.5	77.6	92.3	89.9	81.2	88.4
Sensitivity	99.9	98.04	79.2	100	98.4	76.0	98.7	87.6	61.4	99.7	96.6	73.8
Specificity	8.0	55.2	94.6	7.0	48.5	94.20	16.3	70.4	97.7	14.16	60.6	93.3
PPV	82.4	61.0	71.2	82.61	54.0	70.37	84.0	68.2	82.7	89.8	76.5	78.7
NPV	94.7	97.5	96.4	100	98.0	95.6	73.3	88.7	93.5	88.9	93.0	91.4
F1	90.3	75.2	75.2	90.48	69.7	73.1	90.7	76.7	70.5	94.6	85.4	76.2
C. Kappa (*k*)	0.46			0.41			0.46			0.55		

PPV, positive predictive value; NPV, negative predictive value; F1, F1-score.

### 3.3. Explainability of the model

**Figure 9:**
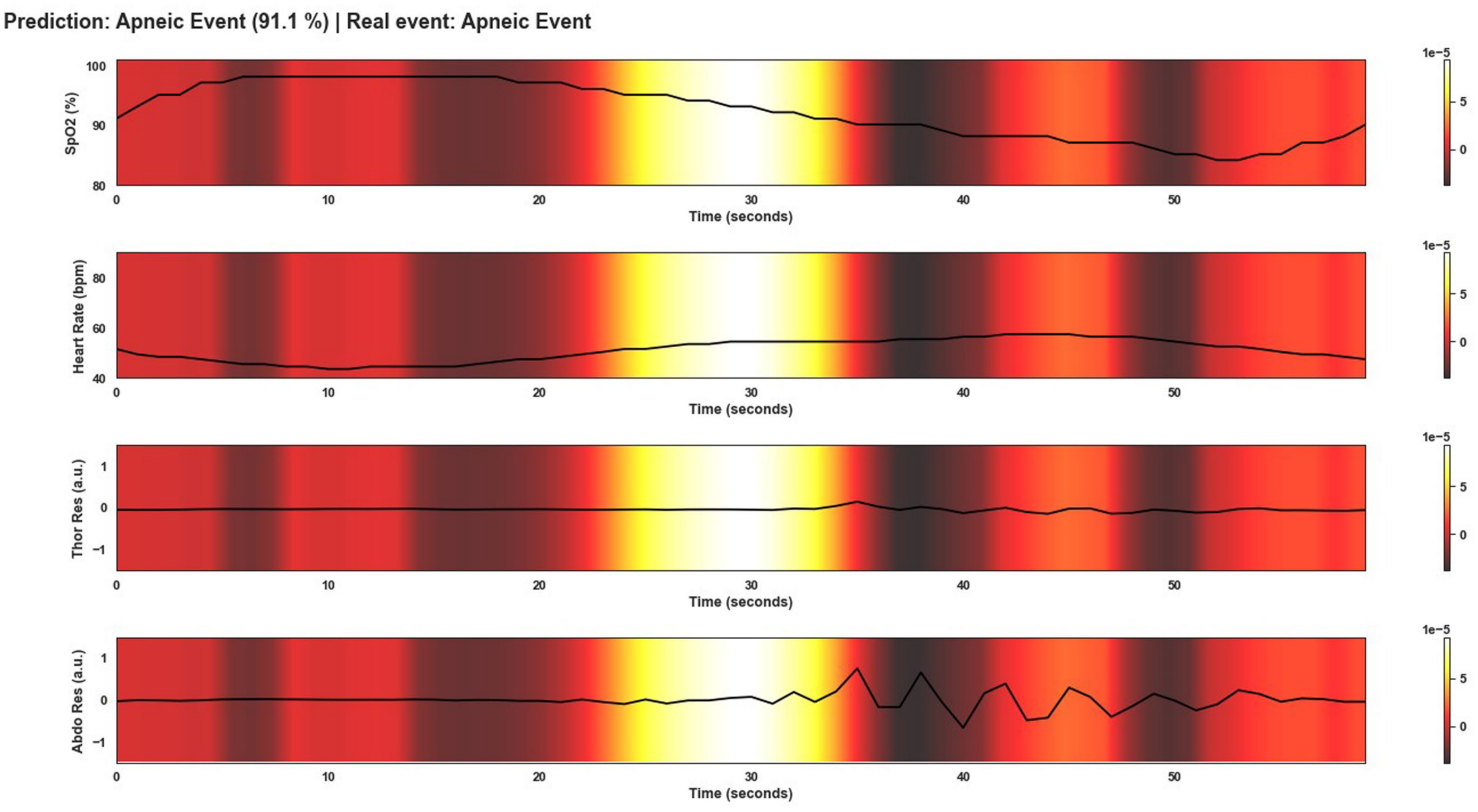
Visualization after application of Grad-CAM to a window correctly classified as an apnea event by Model 4. As can be seen, during the apnea event, SpO2 desaturation occurs and continues until the post-apnea event. On the other hand, the HR response shows a bradycardia and a tachycardia phase in the transition from the apnea event to the post-apnea event. This is typical of an OSA event. For Model 4, the most important region of the signal to classify this window as an apnea event is this change in signal course as can be seen by the lighter area in the middle of the plot.

**Figure 10:**
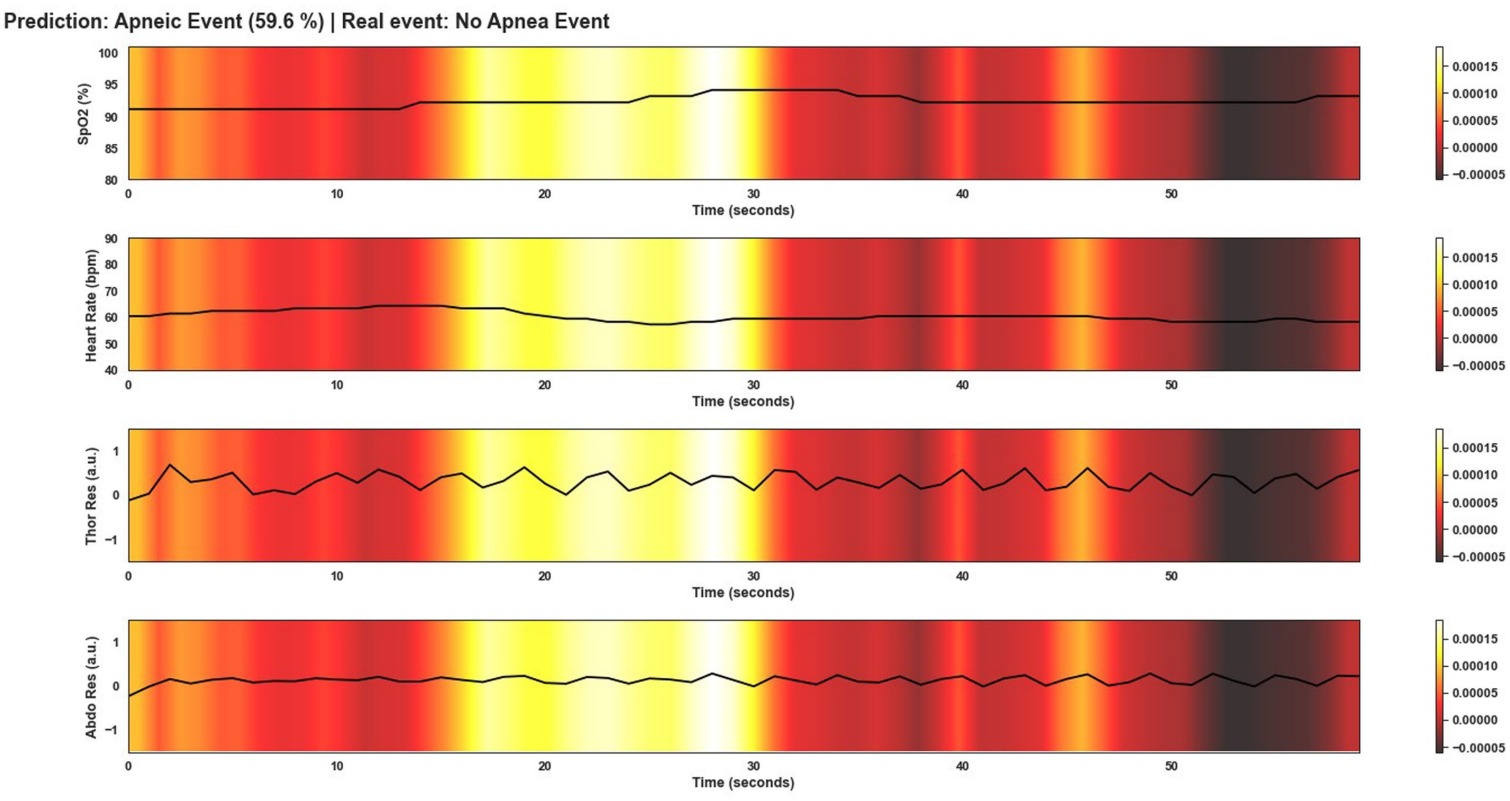
Visualization after application of Grad-CAM to a window incorrectly classified as an apnea event by Model 4. Although the probability of this window being an apnea event is lower than for the window in Figure 9, the event was classified as apnea when in fact, it is not. As can be seen in the area of the signals that are most relevant for the model to make the decision, the changes in SpO2 and HR are the clearest and therefore the most important areas as in Figure 9.

**Figure 11:**
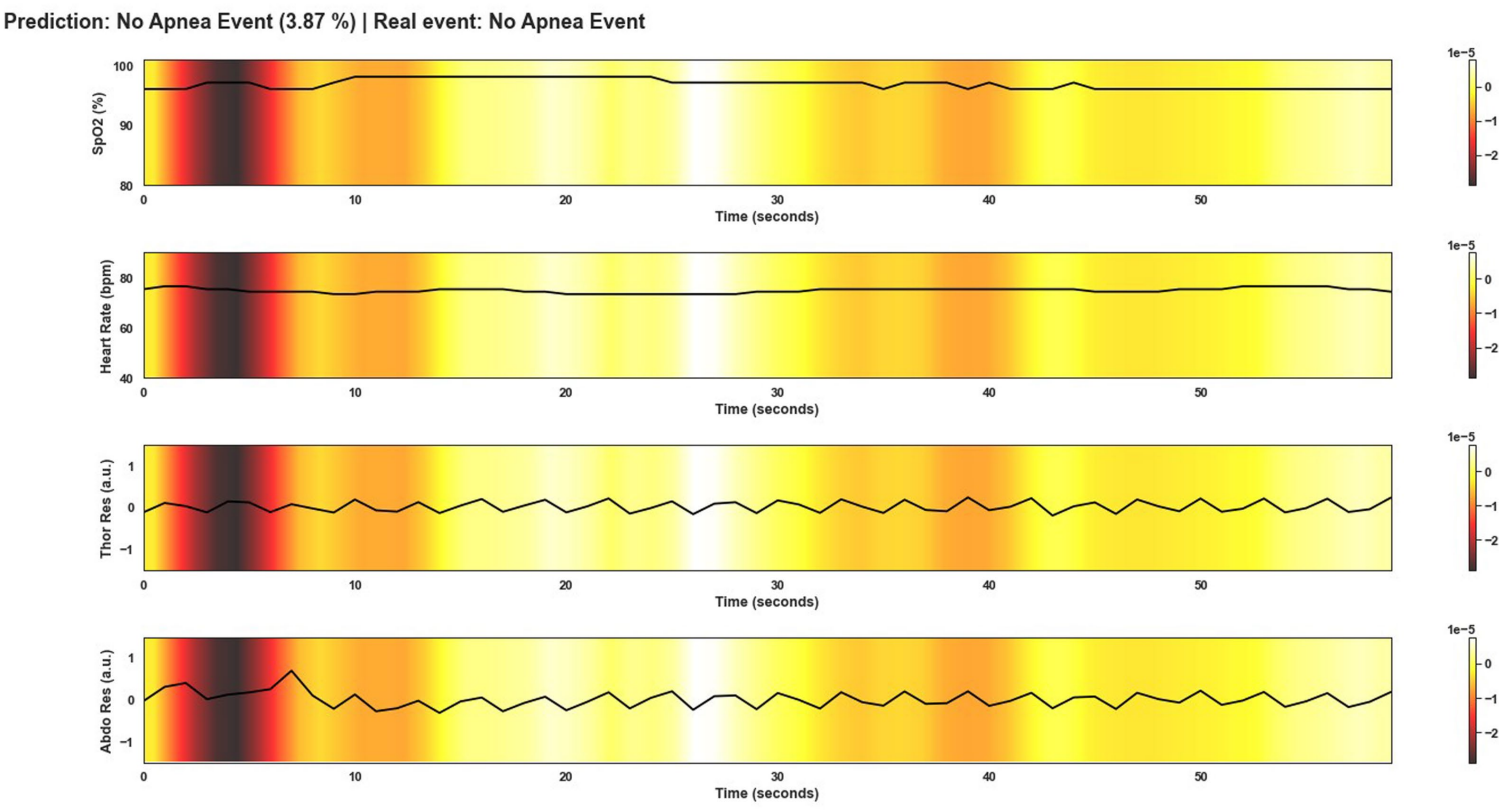
Visualization after application of Grad-CAM to a window correctly classified as non-apnea event by Model 4. As can be seen from the colored areas of the signals, in practice all signal regions are of equal importance to the model. As there are no significant changes in the course of the signals that would indicate an apnea event, unlike in Figure 9, the model predicts this window as not being an apnea event.

**Figure 12:**
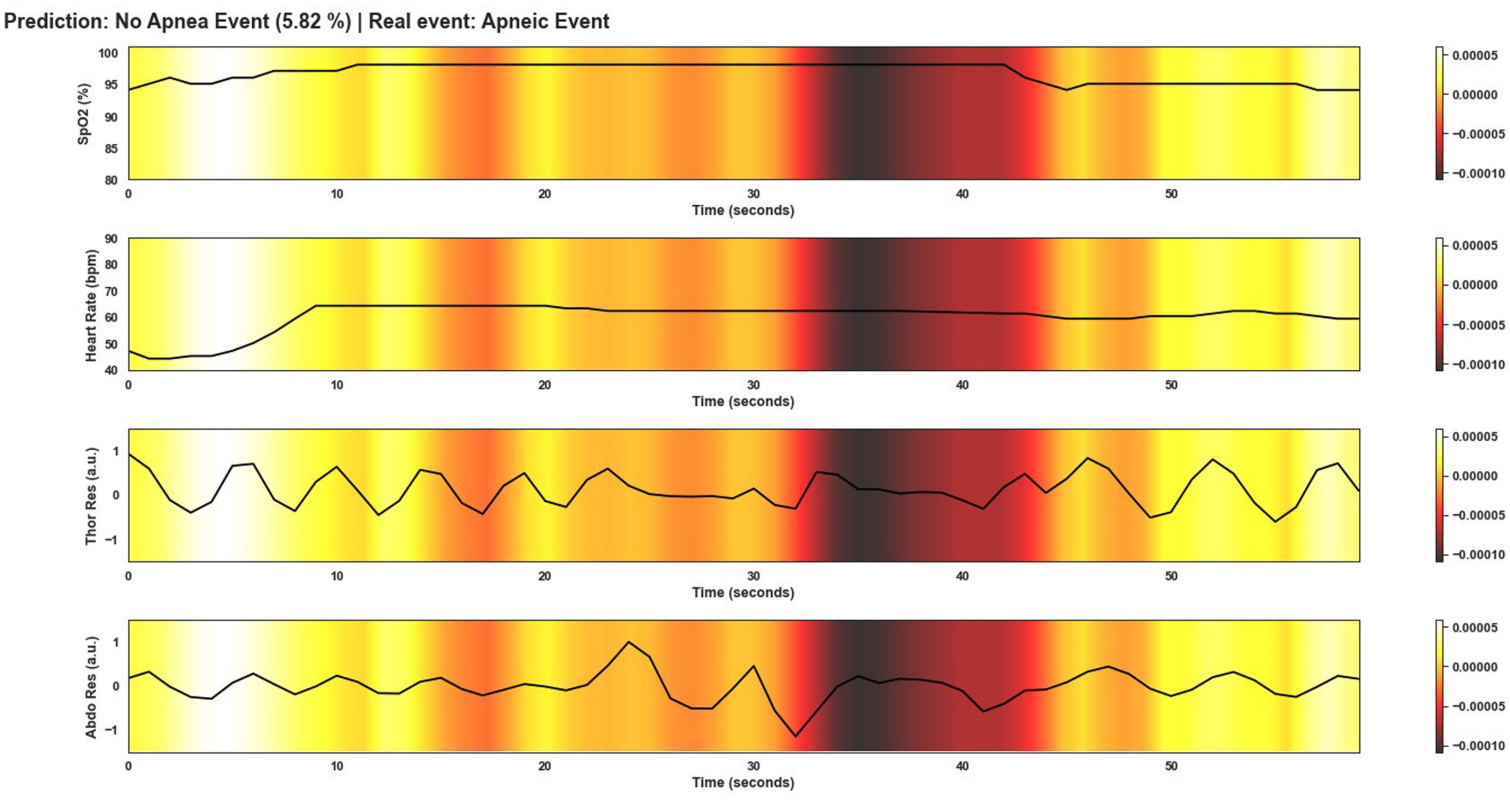
Visualization after application of Grad-CAM to a window incorrectly classified as non-apnea event by Model 4. In this case the Model does not correctly classify the event as apnea. As can be seen in the image, there are no significant changes in the course of the signals, unlike in Figure 9. Only in the first 10 s of the window can abrupt changes be observed. However, this was not sufficient for the model to correctly classify this window as an apnea event. The duration of apnea events also influences the classification.

This section shows several windows after being classified by Model 4. To give an overview of the performance of the model, the events shown correspond to a correctly classified apnea event, an incorrectly classified apnea event, a correctly classified non-apnea event and an incorrectly classified non-apnea event. As can be seen in Figures 9–12, the detection of changes in the course of the signals is essential for a correct classification of the apnea event. Before analyzing the windows after the application of Grad-CAM, it is essential to note that the visualizations show the signal regions that were most relevant for Model 4 to make the decision. This fact does not imply that the regions of the most relevant signals for Model 4 indicate the apnea event itself. In Figure 9, it can be seen the window correctly classified as an apnea event. In this case, the focus is on the HR and SpO2 signals to identify the event. In the peri-apnea phase, it can be seen how the minimum HR causes the minimum SpO2 during the apnea phase. In addition, the maximum HR can be seen in response to the minimum SpO2 during the post-apnea phase. This is a typical HR and SpO2 response during an apnea event. In this first example, the most relevant region for Model 4 is where SpO2 desaturation and HR increase. Thor-Res and Abdo-Res amplitudes remain stable and minimal in a clear apnea event. Therefore, Model 4 learned to identify an apnea event correctly. Figure 10 also shows similar behavior to Figure 9, with small Thor-Res and Abdo-Res amplitudes, a minimum in HR proceeds to desaturation of SpO2. In this case, the SpO2 desaturation is less significant than in Figure 9. Therefore, it is not a desaturation greater than or equal to 3%, which implies that it is not a sleep apnea event. Model 4 recognizes the pattern of an apnea event by looking at the lighter areas, with a decrease in HR, followed by a resaturation, a prelude to the desaturation of SpO2. However, unlike the example in Figure 9, the most relevant signal regions for Model 4 are the decrease in HR and the resaturation of SpO2, when the objective, in this case, would be the detection of the desaturations of SpO2. Figure 11 shows how model 4 considers practically the entire window to be relevant, with the exception of about 5 s at the beginning of the window. As can be seen, no changes in HR and no significant SpO2 desaturation could indicate an apnea event, with SpO2 always greater than 95% throughout the window. Thor-Res and Abdo-Res also show no noticeable changes. Therefore, the use of practically all the signals indicates that for the model, no relevant regions could indicate an apnea event but a non-apnea event in this case. Finally, Figure 12 shows that the regions of the signals most relevant to the model were those with an increase in HR and SpO2 resaturation, in addition to SpO2 desaturation from second 43 onwards. Despite this, the model did not correctly classify this window as an apnea event. Based on these results, it can be stated that windows that include seconds of sleep apnea but do not exceed 10 s in duration or small SpO2 desaturations that are close to 3% or have a small duration, significantly affect the performance of Model 4. In addition, and although less frequent, central apnea or mixed apnea events can also influence the predictive performance of the model.

## 4. Discussion

Even though PSG continues to be the most widely used technique to detect sleep apnea events, PMs are increasingly playing a leading role. It is increasingly common to see PMs that achieve results in terms of accuracy, sensitivity, or specificity that are very similar to those obtained with PSG (Van Steenkiste et al., 2020; Kristiansen et al., 2021). This work exposes the development of a deep learning model that can be implemented in a PM. In addition, this development emphasizes something crucial when aiming to use artificial intelligence algorithms in a real healthcare environment: the explainability of the model. In general, a vast majority of scientific papers focus on getting outstanding metrics performance with machine learning algorithms. In contrast to the proposal shown in this scientific work, such scientific papers focus less on the end user who will use those algorithms: sleep, doctors, or clinicians. A PM should achieve results similar to those obtained with PSG. PMs should also improve numerous drawbacks of polysomnography, such as patient comfort during the sleep test. Since using many sensors, PSG can be considered an invasive technique where sometimes the patient has difficulty falling asleep. On the other hand, PMs are intended to reduce the economic cost of having a sleep clinician during the development of PSG. There are already solutions that can improve these aspects and even improve them. However, the presentation of results and the interpretability of deep learning models using many current solutions still need to provide all the necessary information for the doctor.

This paper showed the results concisely and understandably for a person who is not an expert in machine learning, in addition to providing a guideline to engineers or scientists who work in the field of deep learning to detect sleep patterns. On the one hand, by dividing the patient’s signals into windows of 60 s duration, it is ensured that an apnea event, which can normally last from 10 to 40 s, is included within the window. On the other hand, this implies that loss of information may occasionally arise since an OSA event may be divided by two windows when windowing is performed. However, splitting in this way makes it possible to calculate the AHI, which is a must for doctors. The model presented in this manuscript is trained and evaluated with four physiological signals: SpO2, HR, Thor-Res, and Abdo-Res. These signs were chosen for various reasons. First, this model intends to be used in a real PM, which collects three different signals, including oxygen saturation, heart rate, and impedance (in the absence of impedance data, a combination of Thor-Res and Abdo-Res will have to be used). SpO2 and HR should be signals similar to those used during training. On the other hand, which signal is more suitable to retrain the model once the PM is developed and collects the impedance should be considered. For this reason, the model has been trained with Abdo-Res and Thor-Res, since they are the most similar signals to impedance. As can be seen, the model works with only four signals so that the PM has few sensors and is as comfortable as possible for the patient regarding the chosen deep learning algorithm. Numerous DL models have shown promising results in detecting OSA events (Ramachandran and Karuppiah, 2021). For the development of this work, several of them were considered (Mostafa et al., 2019; Ramachandran and Karuppiah, 2021; JeyaJothi et al., 2022). 1D-CNN was chosen because it offers several benefits in terms of development and subsequent use of the tool by the end user. On the one hand, this type of DL architecture is state-of-the-art and used in many different fields. 1D-CNN has also shown remarkable results in the field of sleep medicine (JeyaJothi et al., 2022). Additionally, its architecture is relatively simple and does not require extensive computational resources to function at full capacity. In addition, it can be a small number of data to yield acceptable results.

On the other hand, it has been mentioned throughout this work that it is essential to keep in mind the role of the physician for developing machine learning models that will be used in a real clinical environment. Considering this, the 1D-CNN are models that can be easily understood by people who are not experts in artificial intelligence. With an adequate technique, it is possible to provide additional tools to make the end user understand, in this case, the doctor, the reason behind a particular decision and not another decision by the model. In this case, the Grad-CAM technique was used to facilitate the interpretability and explainability of the model. This technique has been explained in Section “Explainability of the model”, and the results are also shown in Section “Results”. Using Grad-CAM, it is possible to visualize the most relevant regions of the physiological signal for the model to make the decision. In this case, the model must detect an OSA event, and thanks to Grad-CAM, it is possible to see when it occurs. This tool is also useful to study when an event is correctly classified. This is vital for clinicians.

If a tool with these characteristics is not presented together with the model, it is useless for the model to show promising values for the metrics. In addition, the technique must be understandable to experts in sleep medicine, as there may be occasions when the explainability of the model is so complex that what is widely known as a black box occurs. Thanks to the approach presented in this work, it is possible to detect apnea events with an accuracy of 84.3%, sensitivity of 82.5%, and specificity of 86%. It makes it possible for the DL algorithm to be suitable for working with PMs if we follow the recommendation of some scientific works recommending that PMs must have at least 82.5% sensitivity and positive likelihood ratio (LR+) of at least 5 to be used in a real clinical environment (Collop et al., 2011). In our case we obtained an 82.5% sensitivity and LR+ of 5.89 for the SHHS2 dataset for testing.

Although our aim is to develop a model that can be used in a portable monitor that is also under development. A comparison between the results obtained by our model and other solutions proposed by other authors is shown in Table 6. The scientific papers listed in the table use the same datasets and signals used in this work. However, different sets of signals and a different number of patients were used. As can be seen, our model outperforms many of the solutions shown, and only one proposal achieves better results (Gutiérrez-Tobal et al., 2021). However, this comparison has a particular bias since finding scientific works that use the same workflow to train and test the model is complicated, such as the number of patients, duration of windows, sampling frequency, number of signals used, etc. Moreover, not all solutions claim to use the models on PMs or claim to use visualization techniques to explain the decision made by the model. Furthermore, the number of signals used is also relevant as we consider that using one or two signals, despite achieving good results, is insufficient for the subsequent explainability of the model. Regarding Haidar et al. (2018), there is a relevant difference with respect to our work: Haidar et al. (2018) is based on a multiclass classification with apnea, hypopnea and normal events, while we perform a binary classification. In Haidar et al. (2018) the set of three signals (nasal flow, abdominal and thoracic) gives the best results in terms of accuracy. However, unlike our work, they do not calculate AHI and do not include model explainability. In Van Steenkiste et al. (2019), a distinction is made between OSA, central apnea and hypopnea. AHI calculation is also performed, as in our work, the worst results are obtained with AHI < 5. This overestimation may be due to the fact that the number of events to classify the event as normal is within the lowest range, from 0 to 5, as opposed to the classification of events as mild, moderate or severe, which ranges from 5 to 15, 15 to 30 and more than 30, respectively. In Van Steenkiste et al. (2019), there is also no reference to the explainability of the model. In Haidar and Jeffries (2020) focus more on the explainability of the model. However, unlike our proposal, no visualisation method to study the decision-making of the model is proposed. Biswal et al. (2018) in addition to the classification of apnea events, also developed models for the classification of sleep phases and limb movements. It is similar to our work in the dataset used and the dataset. However, it also does not focus on the interpretability of the model. Finally, Gutiérrez-Tobal et al. (2021) achieved very good results using blood-oxygen saturation signals (SpO2) in predicting OSA events and calculating AHI. It also focuses on the problem of black boxes but without delving as deeply into the subject as our work. In short, besides presenting a deep learning model for apnea event detection and AHI calculation, this work presents a solution for the explainability of the model in a visual way that can be used by end users (in this case, sleep clinicians). In addition, the proposed model is intended to be used on a real portable monitor. This differentiates it from the rest of the work presented in Table 6.

**Table 6:** Comparison of the results obtained by our model with other results obtained by existing models in the scientific literature for OSA detection.

Author	Dataset	Signal	Model	Accuracy	Sensitivity	Specificity	AUC
Haidar et al. (2018)	MESA	Thor-Res Abdo-Res	CNN	77.7%	77.6%	–	–
Van Steenkiste et al. (2019)	SHHS-1	Thor-Res Abdo-Res EDR	LSTM	70%	60.7%	72.8%	72%
Haidar and Jeffries (2020)	MESA	Thor-Res Abdo-Res Airflow	CNN Markov Chain	80.78%	81.73	–	–
Biswal et al. (2018)	SHHS	Thor-Res Abdo-Res Airflow SpO2	RCNN	80.2%	–	–	–
Gutiérrez-Tobal et al. (2021)	SHHS1 SHHS2	SpO2	Least-squares boosting (LSBoost)	89.68% (avg) 88.66% (avg)	87.67% (avg) 94.56% (avg)	79.56% (avg) 64.77% (avg)	–
Our model	SHHS2	SpO2 HR Thor-Res Abdo-Res	1D-CNN	84.3%	82.5%	86%	92.1%

This work presents numerous promising aspects when it comes to the development of deep learning models for apnea detection. The training of the models is done by an engineering approach using Keras Tuner, thus avoiding costly trial and error in time and computational resources. The use of the Grad-CAM technique presents the explainability of the model. In this way, doctors are provided with a method to study the decision made by the model clearly and visually. The AHI is also calculated from the models that obtained the best results during training. Despite all this, the end-to-end solution of DL exposed in this work also includes several limitations. Although the training and test data for SHHS2 and SHHS1 were randomly selected, SHHS2 comprises a subset of SHHS1 patients who participated in a follow-up sleep study 5 years later. Even though the probability is low, this could imply a biased result. The data sets for training and testing contain only a few artifacts or missing values, and the quality of the signals is good. Therefore, Model 4 must be tested in the future on lower-quality data to see its performance. Despite the benefits of choosing 1 Hz for the signal sampling rate, choosing another value for the sampling rate is a fact of interest in future developments of this scientific work. The development of window-fed models entails difficulty avoiding data from the same patient being included in the training, validation and test sets during cross-validation. The cross-validation method and the large number of windows reduce this possibility. However, to facilitate the development of the models, other alternative methods for cross-validation may be applied in the future. It is essential to note the large number of windows used to train the model. Moreover, this windowed dataset was perfectly balanced. This implies that the results are faithful to reality and reduce the bias generated due to the small number of patients used to generate the data for training. Moreover, unbalanced datasets may yield misleading results. Parallel to the development of the PM that will work with the DL model presented in this work, other possible architectures will be addressed to try, on the one hand, to improve the results presented here and reduce or eliminate the limitations described above. One possibility is the use of segmentation models, which have already shown promising results in classifying some sleep pathologies (Perslev et al., 2019). Various limitations of the proposal presented in this paper will also be covered in the future. Firstly, an attempt will be made to calculate the total time in bed instead of total recording time in order to obtain a more accurate AHI result, as the one shown in this paper overestimates the number of apnea events. We will also work with multiclassification tasks in order to distinguish between apnea and hypopnea.

## 5. Conclusion

A 1D-CNN has been developed for the detection of obstructive sleep apnea events. During the development of this model, it has been taken into account that the main objective of the algorithm is to work with a PM to detect sleep apnea. For this, it is essential to consider the figure of the end user of the device, in this case, the doctor or sleep clinician (besides the patient). Therefore, in addition to obtaining optimal results, this work aims to obtain a balance between the accuracy and the explainability of the model. The model can only be used in a medical environment with acceptable interpretability. In addition to providing model explainability, our solution achieved 84.3%, 82.5%, 86%, and 92.1% in terms of accuracy, sensitivity, specificity, and AUC in detecting sleep apnea in SHHS2. The model was also tested on external datasets such as SHHS1 and MESA. Regarding the calculation of the AHI, despite an overestimation of the AHI, promising results were obtained with R2 = 0.65 for the SHHS2 training and test data set. To confirm the reliability of the results, the DL models were tested on both balanced and unbalanced data.

## Data availability statement

Publicly available datasets were analyzed in this study. This data can be found here: https://sleepdata.org/datasets/shhs, https://sleepdata.org/datasets/mesa.

## Author contributions

ÁA, NM, RS, and JO were involved in study design and literature search. ÁA, NM, and RS were involved in validation, data curation, and data analysis. ÁA wrote the manuscript, literature review, and prepared pictures and tables. All authors contributed to the interpretation of the results, manuscript revision and reviewed the final version making the necessary changes, approved the submitted version, and agreed to be accountable for the content of the work.

## Funding

This research was funded by ZiM project “Sleep Lab at Home” (SLaH) grant: ZF4825301AW9 and Carl Zeiss Foundation and the MORPHEUS-Project “Non-invasive system for measuring parameters relevant to sleep quality” (project number: P2019-03-003). The article processing charge was funded by Reutlingen University.

## Conflict of interest

The authors declare that the research was conducted in the absence of any commercial or financial relationships that could be construed as a potential conflict of interest.

## Publisher’s note

All claims expressed in this article are solely those of the authors and do not necessarily represent those of their affiliated organizations, or those of the publisher, the editors and the reviewers. Any product that may be evaluated in this article, or claim that may be made by its manufacturer, is not guaranteed or endorsed by the publisher.
