# Supplementary material for: Obstructive sleep apnea event detection using explainable deep learning models for a portable monitor
Source: Front Neurosci. 2023 Jul 14;17:1155900. doi: 10.3389/fnins.2023.1155900 (PMC10375719; doi:10.3389/fnins.2023.1155900)
Supplement: Supplementary file 1 [file Data_Sheet_1.docx]

Supplementary Material

Obstructive Sleep Apnea Event Detection using Explainable Deep Learning Models for a Portable Monitor

Ángel Serrano Alarcón^1,3*^, Natividad Martínez Madrid^1^, Ralf Seepold^2^, Juan Antonio Ortega Ramírez^3^

^1^ Reutlingen University, Alteburgstr. 150, 72762 Reutlingen, Germany

^2^ HTWG Konstanz, Alfred-Wachtel-Str. 8, 78462 Konstanz, Germany

^3^ University of Seville, San Fernando 4, 41004 Sevilla, Spain

*** Correspondence:**

**Corresponding Author/a**

Angel.serrano_alarcon@reutlingen-university.de

# Supplementary Data

Supplementary Material should be uploaded separately on submission. Please include any supplementary data, figures and/or tables.

Supplementary material is not typeset so please ensure that all information is clearly presented, the appropriate caption is included in the file and not in the manuscript, and that the style conforms to the rest of the article.

# Supplementary Figures and Tables

For more information on Supplementary Material and for details on the different file types accepted, please see [here](https://www.frontiersin.org/guidelines/author-guidelines#supplementary-material).

## Supplementary Tables

| **Tuner** | Hyperband |
| --- | --- |
| **Number of hidden layers** | 4 |
| **Feature map – 1st hidden layer** | 96 |
| **Kernel size – 1st hidden layer** | 3 |
| **Dropout – 1st hidden layer** | 0.1 |
| **Feature map – 2nd hidden layer** | 128 |
| **Kernel size – 2nd hidden layer** | 9 |
| **Dropout – 2nd hidden layer** | 0.5 |
| **Feature map – 3rd hidden layer** | 64 |
| **Kernel size – 3rd hidden layer** | 9 |
| **Dropout – 3rd hidden layer** | 0.3 |
| **Feature map – 4th hidden layer** | 256 |
| **Kernel size – 4th hidden layer** | 11 |
| **Dropout – 4th hidden layer** | 0.4 |
| **Layer Activation (all convolutional layers)** | ReLU |
| **Last-layer activation** | Sigmoid |
| **Learning rate** | 0.001 |
| **Optimizer** | Adam |
| **Loss** | Binary Crossentropy |

Table 1. Set of the best hyperparameters of the Model 1.

| **Tuner** | Hyperband |
| --- | --- |
| **Number of hidden layers** | 4 |
| **Feature map – 1st hidden layer** | 96 |
| **Kernel size – 1st hidden layer** | 3 |
| **Dropout – 1st hidden layer** | 0.3 |
| **Feature map – 2nd hidden layer** | 128 |
| **Kernel size – 2nd hidden layer** | 9 |
| **Dropout – 2nd hidden layer** | 0.4 |
| **Feature map – 3rd hidden layer** | 160 |
| **Kernel size – 3rd hidden layer** | 7 |
| **Dropout – 3rd hidden layer** | 0.2 |
| **Feature map – 4th hidden layer** | 96 |
| **Kernel size – 4th hidden layer** | 7 |
| **Dropout – 4th hidden layer** | 0.2 |
| **Layer Activation (all convolutional layers)** | ReLU |
| **Last-layer activation** | Sigmoid |
| **Learning rate** | 0.0001 |
| **Optimizer** | Adam |
| **Loss** | Binary Crossentropy |

Table 2. Set of the best hyperparameters of the Model 2.

| **Tuner** | Hyperband |
| --- | --- |
| **Number of hidden layers** | 4 |
| **Feature map – 1st hidden layer** | 192 |
| **Kernel size – 1st hidden layer** | 11 |
| **Dropout – 1st hidden layer** | 0.2 |
| **Feature map – 2nd hidden layer** | 128 |
| **Kernel size – 2nd hidden layer** | 9 |
| **Dropout – 2nd hidden layer** | 0.5 |
| **Feature map – 3rd hidden layer** | 160 |
| **Kernel size – 3rd hidden layer** | 9 |
| **Dropout – 3rd hidden layer** | 0.5 |
| **Feature map – 4th hidden layer** | 192 |
| **Kernel size – 4th hidden layer** | 7 |
| **Dropout – 4th hidden layer** | 0.5 |
| **Layer Activation (all convolutional layers)** | ReLU |
| **Last-layer activation** | Sigmoid |
| **Learning rate** | 0.01 |
| **Optimizer** | Adam |
| **Loss** | Binary Crossentropy |

Table 3. Set of the best hyperparameters of the Model 3.

|  | **Model 1** | | | **Model 2** | | | **Model 3** | | | **Model 4** | | |
| --- | --- | --- | --- | --- | --- | --- | --- | --- | --- | --- | --- | --- |
| **Dataset** | **SHHS2** | **SHHS1** | **MESA** | **SHHS2** | **SHHS1** | **MESA** | **SHHS2** | **SHHS1** | **MESA** | **SHHS2** | **SHHS1** | **MESA** |
| Accuracy | 80.50 | 68.6 | 77.9 | 52.8 | 56.1 | 47.3 | 84.7 | 74.16 | 74.1 | 84.5 | 73.9 | 73.2 |
| Loss | 0.43 | 0.70 | 0.50 | 0.77 | 0.74 | 0.95 | 0.36 | 0.60 | 0.73 | 0.36 | 0.58 | 0.74 |
| Sensitivity | 36.5 | 24.9 | 29.3 | 87.6 | 74.0 | 83.7 | 82.7 | 51.6 | 76.3 | 83.6 | 53.2 | 77.0 |
| Specificity | 97.0 | 95.3 | 91.2 | 39.7 | 45.5 | 37.39 | 85.5 | 87.9 | 73.5 | 84.8 | 86.5 | 72.1 |
| Precision | 82.5 | 76.2 | 47.7 | 35.4 | 45.3 | 26.8 | 68.24 | 72.3 | 44.0 | 67.4 | 70.7 | 43.0 |
| AUC | 84.9 | 71.6 | 75.6 | 77.7 | 67.9 | 69.5 | 92.1 | 77.5 | 81.6 | 92.1 | 77.7 | 80.8 |

Table 4. Overall results to evaluate the different 1D-CNN models trained on the unbalanced test datasets (SHHS1, SHHS2 and MESA).
